# Lab-on-chip assay of tumour markers and human papilloma virus for cervical cancer detection at the point-of-care

**DOI:** 10.1038/s41598-022-12557-y

**Published:** 2022-05-24

**Authors:** Benjamin W. Wormald, Nicolas Moser, Nandita M. deSouza, Katerina-Theresa Mantikas, Kenny Malpartida-Cardenas, Ivana Pennisi, Thomas E. J. Ind, Katherine Vroobel, Melpomeni Kalofonou, Jesus Rodriguez-Manzano, Pantelis Georgiou

**Affiliations:** 1 Cancer Research UK Cancer Imaging Centre, Division of Radiotherapy and Imaging, Institute of Cancer Research, London, SW7 3RP UK; 2 Centre for Bio-Inspired Technology, Department of Electrical and Electronic Engineering, Imperial College London, London, SW7 2BT UK; 3 Department of Surgical Oncology, Royal Marsden NHS Foundation Trust, London, SW3 6JJ UK; 4 Department of Pathology, Royal Marsden NHS Foundation Trust, London, SW3 6JJ UK; 5 Department of Infectious Disease, Imperial College London, School of Medicine, St Mary’s Hospital, London, W2 1NY UK

**Keywords:** Cancer screening, Gynaecological cancer, Tumour biomarkers, Tumour virus infections, Cancer screening, Tumour biomarkers, Tumour virus infections, Cancer

## Abstract

Cervical cancer affects over half a million people worldwide each year, the majority of whom are in resource-limited settings where cytology screening is not available. As persistent human papilloma virus (HPV) infections are a key causative factor, detection of HPV strains now complements cytology where screening services exist. This work demonstrates the efficacy of a handheld Lab-on-Chip (LoC) device, with an external sample extraction process, in detecting cervical cancer from biopsy samples. The device is based on Ion-Sensitive Field-Effect Transistor (ISFET) sensors used in combination with loop-mediated isothermal amplification (LAMP) assays, to amplify HPV DNA and human telomerase reverse transcriptase (hTERT) mRNA. These markers were selected because of their high levels of expression in cervical cancer cells, but low to nil expression in normal cervical tissue. The achieved analytical sensitivity for the molecular targets resolved down to a single copy per reaction for the mRNA markers, achieving a limit of detection of 10^2^ for hTERT. In the tissue samples, HPV-16 DNA was present in 4/5 malignant and 2/5 benign tissues, with HPV-18 DNA being present in 1/5 malignant and 1/5 benign tissues. hTERT mRNA was detected in all malignant and no benign tissues, with the demonstrated pilot data to indicate the potential for using the LoC in cervical cancer screening in resource-limited settings on a large scale.

## Introduction

Effective screening for cervical cancer has reduced its incidence and death rate in high-income countries, but worldwide, an estimated 600,000 cases occurred, with 342,000 deaths in 2020. Over 90% of these were in low and middle-income countries (LMICS)^1,2^, where cervical cancer has a $< 50$% 5-year survival rate^3^ compared with $<20$% of high-income countries^1^. The single most important causative factor for the onset of cervical cancer is HPV infection. Integration of the virus genome into the host chromosome^4,5^ allows the viral oncogenes HPV E6 and E7, encoded in the early (E) region of the HPV gene, to promote genomic instability by interfering with centromere duplication during mitosis^3,6,7^. This results in large chromosomal rearrangements and copy number variations. Therefore, HPV DNA primary screening via Hybrid Capture-2 or the G5+/6+ assays, which require either signal amplification or target amplification-based methods using Polymerase Chain Reaction (PCR) and expensive thermocycling equipment, are recognized as an effective method of cervical cancer detection^8^.

Visual inspection of the cervix with Lugols iodine or acetic acid is a potential low cost alternative screening strategy, but is uncomfortable for the patient and suffers from bias associated with visual tests^9,10^. A robust, easily administered point-of-care test using nucleic acid amplification of tumour specific markers multiplexed with HPV would be an invaluable triage tool for cervical cancer detection worldwide. For point-of-care cervical cancer detection, selection of optimal HPV and tumour markers is of critical importance. HPV-16 and HPV-18 have been reported to cause up to 70% of cervical cancer cases, being present in approximately 60% and 10% of cases respectively; occurrence of other prevalent HPV types (which are detected in $<1$–6% of cases worldwide) varies by region^11,12^. Furthermore, proliferative capacity in cancer cells is partly attained through telomere maintenance, and telomerase is universally expressed in HPV-positive cancers^13^. hTERT (human telomerase reverse transcriptase) is a telomerase component that is significantly overexpressed in cervical lesions, being detectable in at least 90% of cervical cancers^14,15^, but presents low to nil expression in normal tissue. Therefore, it is a potentially useful tumour-specific marker.

Loop-mediated isothermal amplification (LAMP) methods have been described for point-of-care detection of DNA and RNA from infectious agents, including SARS-CoV-2^16,17^ and breast cancer markers^18,19^. LAMP was originally developed by Notomi et al.^20^, to allow rapid amplification of nucleic acids at a single temperature, typically between 63 and $65\;{^\circ }$C. The reaction occurs without the need for thermocycling, the technique used in PCR that requires access to specialised equipment. Instead, LAMP relies on auto-cycling strand displacement DNA synthesis conducted by a DNA polymerase with high strand displacement activity, thus making the technique ideal for point-of-care testing. LAMP was originally designed with four primers^20^, however this was extended to six by Nagamine et al.^21^ as this accelerated the reaction. The six primers are: Forward-Inner (FIP), Backward-Inner (BIP), Forward Outer (F3), Backward Outer (B3), Loop Forward (LF) and Loop Backward (LB). A stem-loop structure is formed via the binding of the FIP to the target region and the hybridisation of the F3 primer to its target region, forming the stem-loop and allowing strand displacement DNA synthesis to begin. Similarly, the BIP and B3 primers bind on the other end of the target sequence, forming a ‘dumbbell like’ structure^20^. Subsequently, an exponential generation of inverted repeats is constructed as the inner primers anneal and cause amplification from the loops in the original structure. The loop primers, LF and LB, allow hybridisation to the available stem-loops which are not hybridised by the inner primers (FIP/BIP). This significantly accelerates the reaction from 1 h to 10–15 min depending upon the concentration of the starting material. LAMP exhibits high specificity due to the use of four to six primers recognizing six to eight distinct regions, with FIP and BIP playing crucial roles in the specificity of the assay.

LAMP primers for HPV-16/18 DNA used alongside hTERT and GAPDH (used as a positive control) mRNA primers are transferable to the LoC device that reports results via Bluetooth to a smart phone. The device is based on Complementary Metal-Oxide-Semiconductor (CMOS) technology coupled with Ion-Sensitive Field-Effect Transistor (ISFET) sensors. This allows the LoC to detect changes in pH that occur during nucleic acid amplification, in which hydrogen ions are released during nucleotide incorporation. LAMP technology on a LoC was first described by Hu and Georgiou et al.^22^ and has been applied to the detection of breast cancer biomarkers^18,19,23^ and infectious diseases diagnostics including COVID-19^17^. The purpose of this study, therefore, was to translate a LAMP technique using optimally designed primers for HPV DNA and hTERT mRNA to a LoC device and test its ability to detect the presence of cervical cancer, in a pilot feasibility study.

## Results

**Figure 1:**
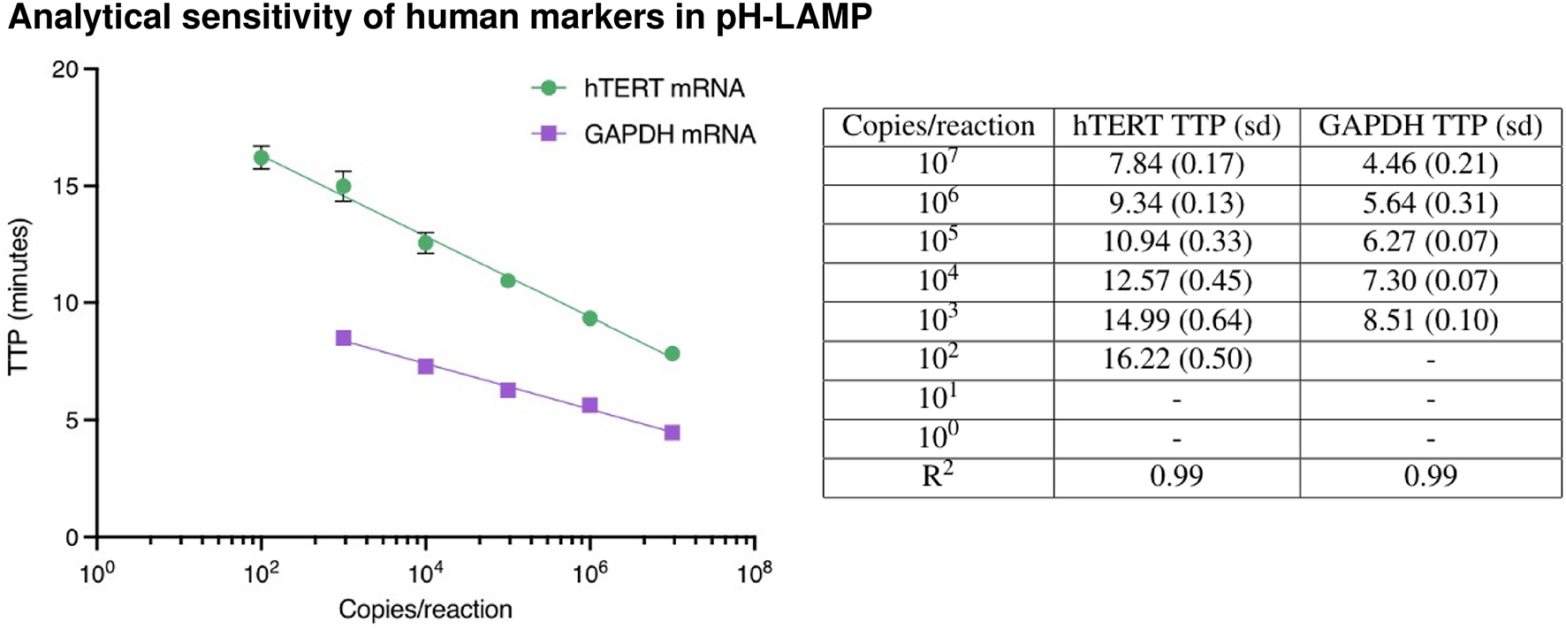
TTP in minutes of serial dilutions of hTERT and GAPDH synthetic mRNA pH-LAMP reaction with respective error bars and results.

The LAMP assays for hTERT and GAPDH mRNA were tested with ten-fold serial dilutions of synthetic RNA ranging from 10^7^ to 10^0^ copies per reaction. For quantification, the time to positive (TTP) values were obtained using the cycle-threshold metric, Ct, set at 0.2 normalized fluorescence units by the LC96 instrument. Standard curves from these results are shown in Fig. 1 with high linearity (R^2^ = 0.99) and a limit of detection of 100 and 1000 copies/reaction and maximum TTP of less than 20 and 10 min, respectively.

### Analytical sensitivity of human markers in pH-LAMP

Validation of HPV DNA and hTERT mRNA using the LOC was done in 10 of 45 available samples from a local tissue collection in which DNA and RNA quantity and quality indicated excellent extraction due to the high number of cells. Of these 10 cervical biopsy samples, there were 5 benign, 4 squamous cell carcinoma (SCC) and 1 adenocarcinoma (ADC) classified on pathology (by K.V. with $>5$ years experience as a specialist gynaecological histopathologist).

Using the LAMP mix on the LC96 thermocycler, HPV-16 DNA was detected in samples H5, H7, H17 and H27, whereas HPV-18 DNA was detected in samples H11 and H45. The LoC platform successfully confirmed detection, with all results being summarised in Table 1. Two cases (H2 and H12) were negative for HPV-16 DNA on the LC96 device using pH-LAMP but were positive when measured on the LoC platform. The presence of HPV-16 DNA in these samples was confirmed using PCR primers. To explain this discrepancy, it may be the case that the copy number of HPV-16 DNA in both of these samples was at the limit of detection or using the same mix/primer regime on both devices yields a more sensitive performance using the LoC platform or that the larger sample volume on the LoC enabled the detection of lower copy numbers at the edge of the detection limit. The presence of hTERT mRNA was detected in 5/5 of cancer samples and 0/5 of benign samples, perfectly delineating the benign from the tumour samples, with results confirmed using the LoC platform. The principle of using a highly sensitive initial test, such as HPV DNA, as well as a secondary triage test with high specificity is appropriately demonstrated here and the near perfect agreement between systems indicates the potential of this platform for further testing. The time to positive (TTP) of less than 25 min on the LoC platform for all tests demonstrates the true point-of-care potential of this system to deliver rapid, accurate results on a portable platform.

**Table 1:** Summary table of Nanodrop DNA and mRNA concentration, TTP and standard deviation (sd) or cycle threshold (Ct) for pH-LAMP or PCR test on LC96 system and LoC platform. *HPV-18 DNA was detected.

Sample name	Diagnosis	DNA conc. (ng/$\upmu$L)	RNA conc. (ng/$\upmu$L)	GAPDH RNA TTP (sd)	HPV-16/18* DNA TTP (sd)	HPV-16/18* LOC TTP	HPV-16/18* PCR Ct (sd)	hTERT RNA TTP (sd)	hTERT LOC TTP	hTERT PCR CT (sd)
H2	Benign	322.30	22.80	9.02 (0.11)	–	17.65	35.51	–	–	–
H5	SCC	1745.00	1434.40	7.06 (0.11)	20.56 (0.56)	15.57	21.41 (0.02)	12.42 (0.34)	21.38	33.48 (0.03)
H7	SCC	1811.80	1576.50	5.70 (0.05)	18.87 (0.23)	13.65	35.95 (0.40)	12.83 (0.77)	21.72	33.70 (0.69)
H9	Benign	590.30	195.40	6.96 (0.04)	–	–	–	–	–	–
H11	Benign	169.50	44.40	7.96 (0.16)	12.48* (0.49)	7.42*	27.16* (0.11)	–	–	–
H12	SCC	1412.70	718.60	6.69 (0.13)	–	17.48	36.30 (0.12)	14.21 (2.79)	18.67	31.57 (0.09)
H17	Benign	2192.80	957.40	8.67 (0.12)	35.40 (5.08)	15.40	34.81 (1.93)	–	–	–
H18	Benign	22.70	9.20	9.20 (0.09)	–	–	–	–	–	–
H27	ADC	1510.50	1570.90	7.97 (0.05)	20.74 (0.26)	18.43	32.61 (0.31)	15.06 (4.01)	14.77	32.75 (0.30)
H45	SCC	1439.30	1048.20	6.47 (0.07)	18.63* (0.21)	18.60*	29.73* (0.02)	14.82 (2.22)	8.88	32.06 (0.33)

## Discussion

This pilot feasibility study has demonstrated the capability of a LoC device to accurately detect the presence of cervical cancer from cervical tissue biopsies using LAMP, specific to HPV DNA and hTERT mRNA as a triage marker, but also nucleic acid markers of both HPV infection and the host genomic consequences of persistent HPV effect resulting in malignancy. This allows both high sensitivity and specificity, bypassing the issue of tests that detect solely HPV DNA which can have high rates of false positives, if not performed in conjunction with other screening tests^24^. Furthermore, HPV DNA screening methods are dependent on expensive equipment which can be inaccessible at locations worldwide, particularly LMICs, most affected by cervical cancer. Other screening methods, such as HPV “self-test” kits, are based on detection of antibodies to HPV strains and primarily detect high-risk strains, which are dependent on the host immune reaction to the presence of the HPV and may be falsely negative, especially in the acute phase of the infection.

Electrochemical systems with chip based biosensors have also been reported for detection of HPV DNA. One such system has been shown to use 16 gold working electrodes arranged in a four by four distribution on a borosilicate glass chip measuring $21\,{\hbox {mm}} \times 23\,{\hbox {mm}}$^25^. Each working electrode is placed between a silver pseudo reference and a gold counter electrode of the same size in order to create 16 planar electrochemical cells. The assay is based on co-immobilisation of HPV E7 and E6 thiolated probes with backfiller, a hybridisation process and electrochemical detection. The limits of detection are described in the pM range and are sufficient for most real RNA/DNA samples, but the system is bulky and not easily adaptable to population screening. In another system, target HPV DNA is captured via magnetic bead-modified DNA probes, followed by an antidigoxigenin-peroxidase detection system at screen-printed carbon electrode chips, with eight samples measurable simultaneously^26^. In comparison, the system described in this paper uses a LoC cartridge that performs on-chip real-time DNA amplification and is able to detect viral DNA by LAMP. This simplifies readout of DNA/RNA amplification and is also potentially adaptable to high throughput, as required for screening applications.

DNA validation was performed on HPV and similarly RNA validation was performed on hTERT. hTERT was selected as previous literature has suggested its role as a promising tumour marker in cervical cancer due to the fact that it is not expressed in normal tissue^14,15,27,28^.

The analytical sensitivity of the selected markers was more than adequate for our purposes. The mRNA markers had a poorer sensitivity compared to the DNA markers, with a limit of detection of 10^2^ for hTERT, and are comparable to other LAMP-based markers. Given that the average quantity of extracted total RNA in archived cervical samples is 27.5 ng/$\upmu$L^29^, the limit of detection of 10^3^ copies per reaction for mRNA is several orders of magnitude lower than the one found in normal clinical samples. Further, GAPDH is present in abundance thus a limit of detection of 10^3^ per reaction is adequate in order for it to function as a positive control. Nevertheless, the limitations of pursuing goals of ultra-sensitivity with these assays comes at the price of loss of specificity and can lead to patients classified as positive, who in truth have no clinically relevant disease. Therefore, a balance between optimal sensitivity without increasing the number of false positive classifications, which would require further (unnecessary) investigations, needs to be made.

Our study confirms that the assay was able to differentiate benign from tumor on a standard benchtop PCR machine with equivalent results on the LOC device for solid tumor tissue. We acknowledge however, that in a screening setting, the samples used would be cytological rather than solid tissue biopsy. Although the amount of tumor tissue available from a biopsy sample is greater than from a cytological swab sample, the latter method yields upwards of 50,000 cells, which is more than adequate for extracting detectable amounts of mRNA. Moreover, cell lysis and DNA/ mRNA extraction is much easier from a cytological swab as homogenization of the cellular material is not required. Therefore, the performance of the designed markers seems to sit squarely in the clinically relevant zone andshould be applicable when using brush cervical cytology samples.

Validation of the HPV and hTERT LAMP reactions against conventional PCR showed largely equivalent results for both the DNA and RNA. Validation of the clinical testing of hTERT and HPV-16 against standard PCR was also equivalent. The LoC system was in close agreement with the traditional benchtop LC96 system, which indicates the potential of this platform as a diagnostic tool.The sensitivity and specificity of the LOC for detecting CIN needs to be explored in future work. Detection of HPV DNA using this assay would potentially identify all women with CIN and cancer, whilst the addition of hTERT mRNA could be employed as a cancer-specific marker. A large prospective study utilizing the assay on a chip at point of care in a screening population is essential to establish its role in this setting.

## Methods

### Primer design

hTERT primers were designed to avoid amplification of the two main splice variants, alpha and beta, which are inhibitory to telomerase function. Primers for hTERT and GAPDH mRNA were designed and selected based on their analytical sensitivity, which was then further documented using a LAMP technique. Together with HPV-16/18 DNA primers designed by Luo et al.^30^, they were subsequently transferred to a specially designed LOC platform, where they were used to detect hTERT mRNA and HPV DNA extracted from tissue samples taken from women with a diagnosis of cervical cancer.

The genomic sequences for hTERT (GenBank Accession number NG_009265.1, NM_198253.2, NM_001193376.1, NR_149162.1, NR_149163.1) were retrieved from the National Institute of Health GenBank system and visualised with Geneious software. Within Geneious the sequences were aligned with the MUSCLE method (MUltiple Sequence Comparison by Log-Expectation)^31^. The mRNA transcript variant sequences were also downloaded. LAMP primers were designed using Primer Explorer V5 (http://primerexplorer.jp/lampv5e/index.html). The splice variant locations for TERT^32^ were added to the sequences prior to primer design.

**Figure 2:**
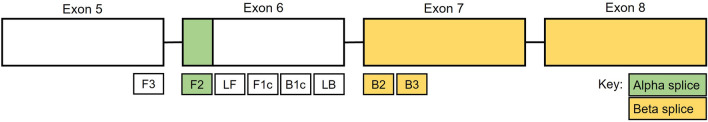
Primer locations on hTERT mRNA. FIP is comprised of F1c and F2, BIP of B1c and B2.

Within hTERT, there are multiple transcript variants but only the full length mRNA transcript conserves telomerase activity^33^. Therefore, exclusion of the two main variants, alpha and beta, that are inhibitory to telomerase function, was necessary. The alpha splice variant removes the first 36 bp of exon 6, while the beta splice variant removes 182 bp from exons 7 and 8^32^. As the F2 primer is located within the 36 bp alpha splice sequence and the B2 and B3 primers are located in the portion of exon 7 which is part of the beta splice, a primer set specific to full length mRNA, that was unable to amplify the main inhibitory splice variants and was exon–exon spanning was designed; this is depicted in Fig. 2.

### Primer selection

Primer selection based on analytical sensitivity used synthetic DNA samples for validation. Synthetic double-stranded DNA containing the sequence of the target DNA or RNA (Integrated DNA Technologies, Leuven, Belgium) was quantified using a Qubit 3.0 fluorometer and high-sensitivity double-stranded DNA assay kits (Life Technologies, Carlsbad, CA) on a LightCycler 96 System (Roche Molecular Systems, Pleasanton, CA). The hTERT primers were tested against a synthetic sequence specific to alpha and beta splice variants. The best primer sets were selected as follows:

Step 1 Primer sets for RNA based markers were tested against human genomic DNA (Thermo Fisher Scientific) to ensure the primers were specific to RNA, and not DNA.
Step 2 All primer sets were tested against a synthetic double-stranded DNA or RNA containing the sequence of interest, including appropriate non-template controls. In this initial primer screen the synthetic sequence was diluted to a 10^6^ copy/reaction.
Step 3 The two primer set candidates that achieved the fastest TTP were then tested in a dilution screen. Analytical sensitivity of the assays was evaluated in serial 10-fold dilutions of synthetic DNA or RNA target. This was performed using either 10^8^–10^0^, 10^6^–10^0^ or 10^4^–10^0^ serial fold dilutions to select the most sensitive of the two primer sets candidates.

**Table 2:** LAMP and PCR primer sequences for hTERT and GAPDH mRNA, in $5^{\prime }$–$3^{\prime }$ direction.

Primer	hTERT	GAPDH
F3	GCCTGAGCTGTACTTTGTCA	GATGCTGGCGCTGAGTAC
B3	GGTGAGCCACGAACTGTC	GCTAAGCAGTTGGTGGTGC
FIP	TGGGGTTTGATGATGCTGGCGAGGGCGCGT ACGACACCATCC	CTTTTGGCTCCCCCCTGCAAATGGAGTCC ACTGGCGTCTT
BIP	GGTCCAGAAGGCCGCCCATGCTGGAGGTCT GTCAAGGTA	TCTGCTGATGCCCCCATGTTCGGAGGCAT TGCTGATGATCT
LF	ACCTCCGTGAGCCTGTCCTG	AGCCTTCTCCATGGTGGTG
LB	CACGTCCGCAAGGCCTTCA	GTCATGGGTGTGAACCATGAG
PCR—forward	TCAAGGTGGATGTGACGGG	GGGAAGGTGAAGGTCGGAGT
PCR—reverse	GGACTTGCCCCTGATGCG	TGGAAGATGGTGATGGGATTTC

### LAMP reaction conditions

The pH-LAMP mix for HPV DNA test contained the following: 2.4 $\upmu$L of Betaine (stock at 5 M), 1.5 $\upmu$L of customised isothermal buffer (pH 8.5–9), 0.9 $\upmu$L of MgSO4 (stock at 100 mmol/L), 0.9 $\upmu$L of bovine serum albumin (20 mg/mL), 0.84 $\upmu$L of dNTPs (stock at 25 mmol/L), 0.375 $\upmu$L of Syto9 (20 mmol/L stock), 0.375 $\upmu$L of sodium hydroxide (0.2 M), 0.04 $\upmu$L of Bst 2.0 DNApolymerase (120,000 U/mL) (New England Biolabs, Hitchin, UK), 1.5 $\upmu$L of $10\times$ primer mixture (20 mmol/L BIP/FIP, 10 mmol/L LB/LF, and 2.5 mmol/L B3/F3), 3 µL of synthetic DNA (Integrated DNA Technologies) template solution, and enough nuclease-free water to bring the volume to 15 $\upmu$L. The pH-LAMP mix for hTERT and GAPDH mRNA tests contained the same reagents as the HPV DNA test, in addition to 0.375 $\upmu$L of SuperScript${\circledR }$ III RT/Platinum$^{\circledR }$ Taq (Thermo FisherScientific, Waltham, USA), 0.15 $\upmu$L of ${\hbox {RNaseOUT}}^{\hbox {TM}}$ Recombinant Ribonuclease Inhibitor (Thermo FisherScientific, Waltham, USA) and enough nuclease-free water to bring the volume to 15 $\upmu$L.

### PCR reaction conditions

PCR was used as the ’gold standard’ method against which the efficacy of the newly developed LAMP assay was validated. The PCR primers used are displayed in Table 2. The reaction conditions were based on the conditions laid out in Wang et al.^27^.

### Determining the analytical sensitivity of selected primers using a LAMP technique

For the RNA markers, synthetic double-stranded DNA containing a T7 promoter for RNA transcription synthesised by Integrated DNA Technologies (Leuven, Belgium) was used. The HiScribe T7 High-yield RNA synthesis kit (New England Biolabs, Hitchin, UK) was used to produce target specific RNA to test the designed primers. The synthetic DNA and RNA was quantified using a Qubit 3.0 fluorometer and high-sensitivity double-stranded DNA and single stranded RNA assay kits (Life Technologies, Carlsbad, CA). Sensitivity of the assay was evaluated in serial 10-fold dilutions of synthetic DNA/RNA target, ranging from 10^8^ to 10^0^ copies per reaction with each condition run in triplicate. pH-LAMP assays were performed on the LightCycler 96 System and data analysed using the system software version SW1.1.

### Transferring the LAMP technique to the LoC platform

**Figure 3:**
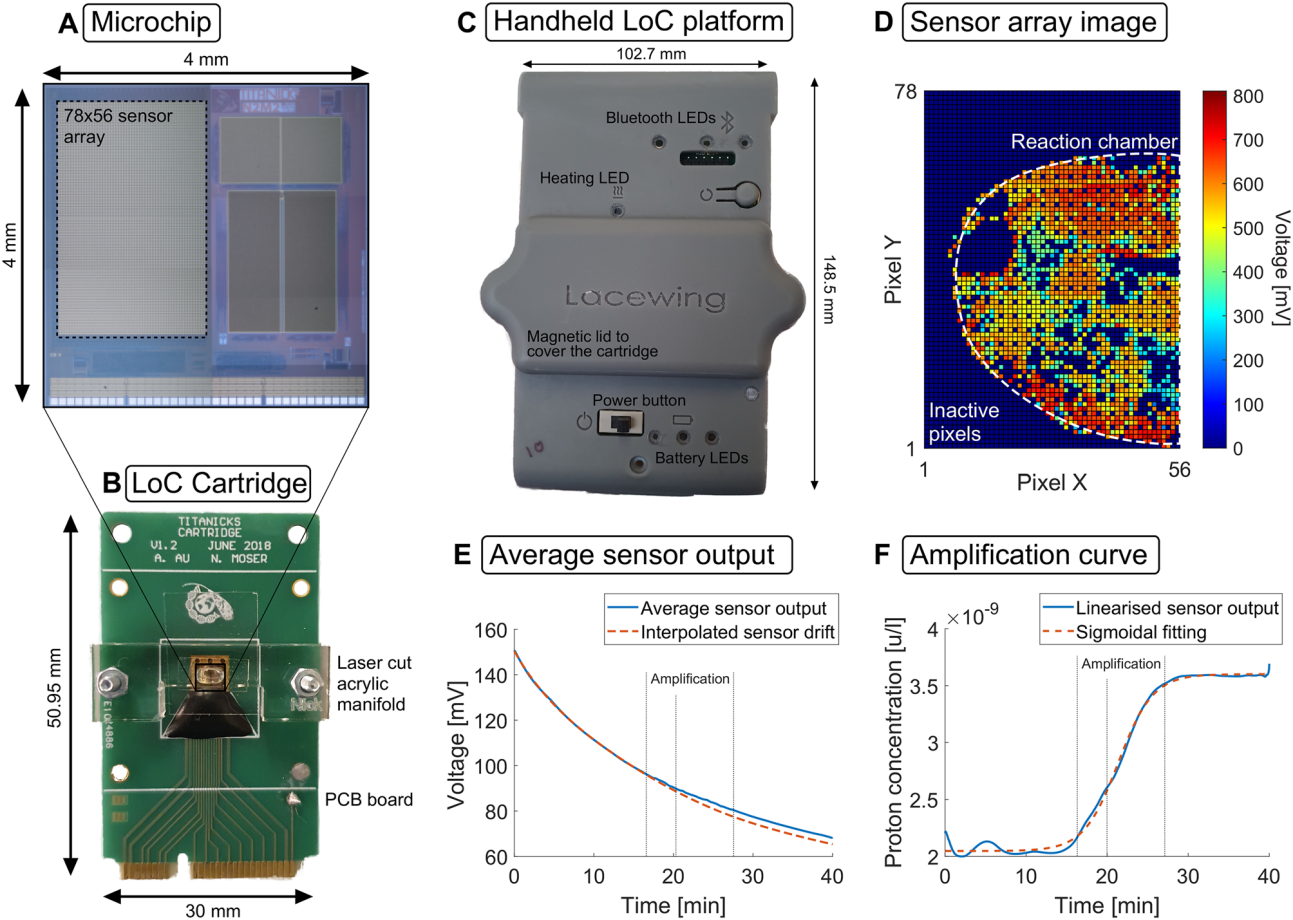
Illustration of the LoC setup and the data processing methodology. (**A**) Microphotograph of the TITANICKS microchip illustrating the $78 \times 56$ electrochemical sensor array, (**B**) LoC PCB-based cartridge with laser cut acrylic manifold to form the reaction chamber, (**C**) handheld *Lacewing* platform for temperature regulation, data acquisition and Bluetooth transmission, (**D**) initial sensor array frame for sample H27, illustrating the spatial imaging capabilities of the sensor array, (**E**) average sensor output with interpolated sensor drift and (**F**) final amplification curve obtain with sensor output linearisation and sigmoidal fitting.

The LAMP assay was translated to a portable LoC platform. This utilises a cartridge which integrates standard and low cost components including a state-of-the-art microchip, a Printed Circuit Board (PCB) and a microfluidic manifold (Fig. 3). The microchip is implemented in a standard CMOS process which is ideal for scalability and low cost. It includes a large array of $78 \times 56$ ISFETs for large-scale potentiometric sensing to monitor nucleic acid amplification during LAMP through the release of protons during nucleotide incorporation. The increase in proton concentration is proportional to the total number of nucleotides inserted and inversely proportional to the buffering capacity of the solution. Therefore, DNA amplification modifies the overall pH of the solution causing a change in voltage detected by the ISFETs. When no amplification occurs, the pH of the solution remains unchanged therefore giving a constant voltage signal. The reaction mix of a standard LAMP therefore was modified to pH-LAMP^34^ by adjusting the buffering capabilities to allow for changes in pH to be measured during DNA amplification.

The CMOS microchip (Fig. 3A) was attached to a printed circuit board (PCB) and a laser cut acrylic manifold was bolted on top to form a sealed reaction chamber for the reaction. The manifold was designed for a reaction volume of 20 $\upmu$L and sealing between the manifold and chip surface was accomplished through laser cut double-stick Tessa adhesive gaskets. An Ag/AgCl reference electrode (chloridised 0.03 mm Ag wire) was placed between the chip surface and the tape gasket. The cartridge is shown in Fig. 3B. Reactions were performed for 30 min, and data was recorded in situ in real-time. The LoC device (*Lacewing*) was designed as a handheld platform to perform heating and temperature regulation for the LAMP assay, data acquisition from the microchip, and Bluetooth transmission to a smartphone app developed on AndroidOS (Fig. 3C). Temperature regulation was performed with a PID controller on the device microcontroller using on-chip temperature sensors and a Peltier module placed in contact with the bottom side of the cartridge. The device is powered using a battery integrated at the bottom of the 3D printed case. Spatial monitoring of the reaction pH (Fig. 3D) was averaged over all active sensors and processed in MatLab to interpolate and cancel sensor drift (Fig. 3E)^35,36^. The output was linearised and then normalised in order to provide results proportional to the number of nucleotides in solution corresponding to the amplification curve (Fig. 3F). The TTP was then extracted based on cross-thresholding at 0.2 normalized fluorescence units.

### Validation of LoC technology using cervical tissue samples

Tissue samples from cervical cancer patients, from which adequate DNA and mRNA was extracted, were tested using the LAMP technique on the LoC in order to test its ability to detect HPV DNA/RNA and hTERT mRNA as biomarkers for the presence of cervical cancer. Furthermore, to confirm the presence of high quality mRNA obtained from the extractions, GAPDH (glyceraldehyde-3-phosphate dehydrogenase, a common ‘housekeeping’ gene {hlwhich is used as a positive control) mRNA primers were designed and LAMP assays were performed, prior to using the mRNA for hTERT assays.

An existing holding of 45 cervical tissue biopsies were used, collected in 2001 from patients treated at Hammersmith Hospital, London, with their informed consent, and stored at $-80\,{^\circ }$C. The use of these samples for our project was reviewed and approved by London-Surrey Research Ethics Committee Ref: REC18/LO/0865 and performed in accordance with the Declaration of Helsinki. Pathological verification of cancer was undertaken by splitting the sample in half and using half for DNA/RNA extraction and the other half for histological evaluation.

For DNA/RNA extraction, the tissue was homogenized in 2 mL tubes containing RLT+/BME lysis buffer solution from the AllPrep DNA/RNA Kit (Qiagen, Hilden, Germany) and zirconium oxide beads in a Precellys 24 Homogenizer (Bertin, Villeurbanne, France). DNA and RNA were extracted using the AllPrep kit, according to the manufacturer’s instructions. Total DNA and RNA yield was determined using a NanoDrop ND-2000 spectrophotometer (ThermoFisher Scientific, Waltham, USA). Only those samples which yielded both DNA and RNA were selected for testing. Testing was undertaken on the LOC platform and a comparison made with a pH-LAMP experiment on the LC96 system. For histological evaluation, paraffin embedded sections were mounted on glass slides and stained with haemotoxylin and eosin. Slides were reviewed by a specialist consultant gynaecological histopathologist, who classified the sample as benign or malignant tissue (“Supplementary information”).

## Supplementary Information

Supplementary Figures.
