# Supplementary material for: Lab-on-chip assay of tumour markers and human papilloma virus for cervical cancer detection at the point-of-care
Source: Sci Rep. 2022 May 24;12:8750. doi: 10.1038/s41598-022-12557-y (PMC9128326; doi:10.1038/s41598-022-12557-y)
Supplement: Supplementary file 1 — Supplementary Figures. [file 41598_2022_12557_MOESM1_ESM.pdf]

Supplementary Information

Additional Lab-on-Chip data with updated sigmoidal fitting values for each of the clinical samples tested, as originally presented in Table 1 of the manuscript. Figures S1-13 correspond to the samples H2, H5, H5-hTERT, H7, H7-hTERT, H11, H12, H12-hTERT, H17, H27, H27-hTERT, H45, H45-hTERT respectively.

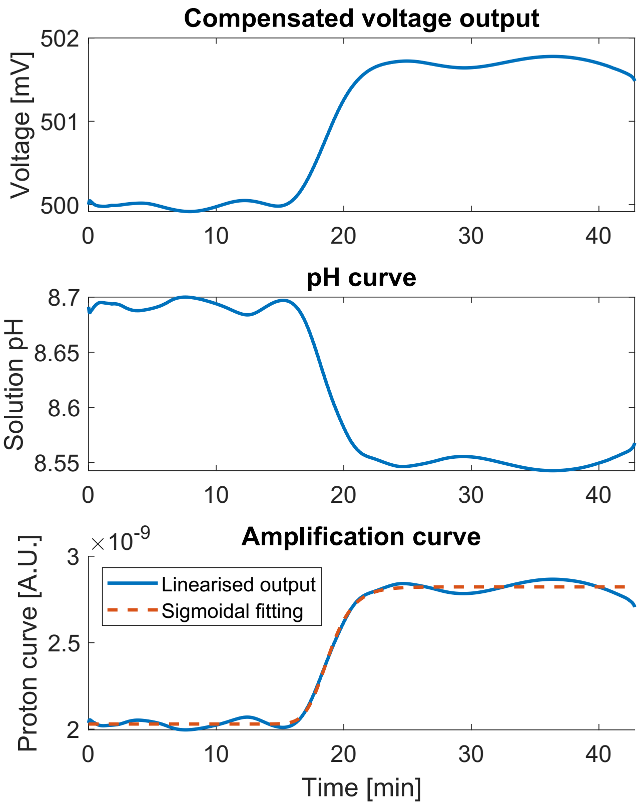

Figure S1. H2.

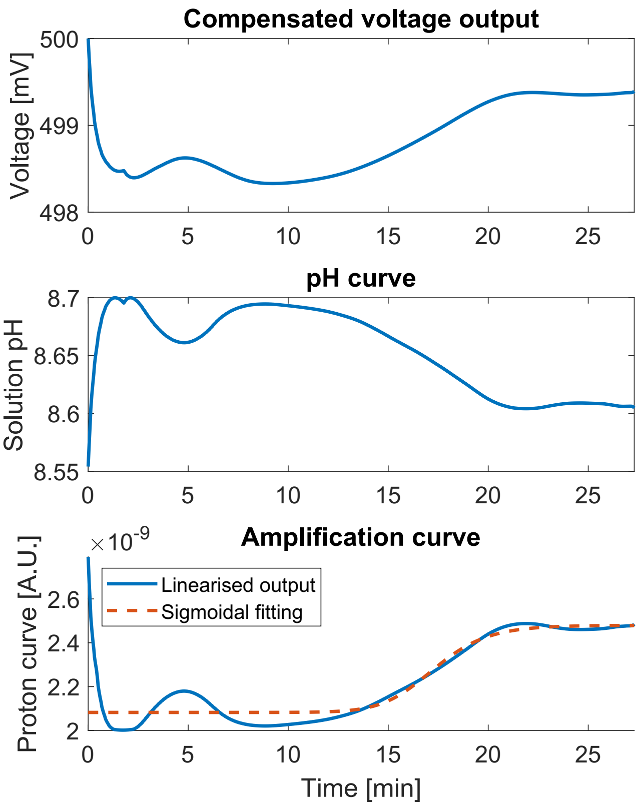

Figure S2. H5.

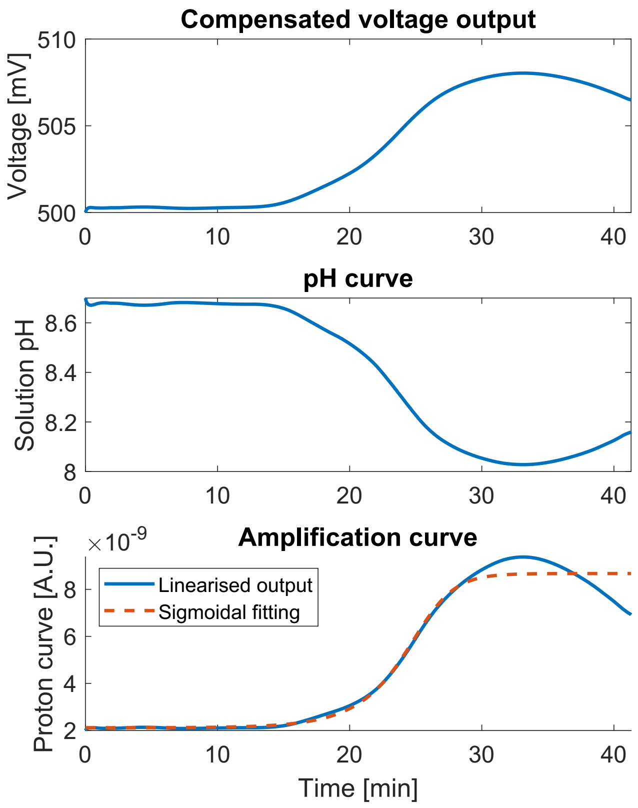

Figure S3. H5 hTERT.

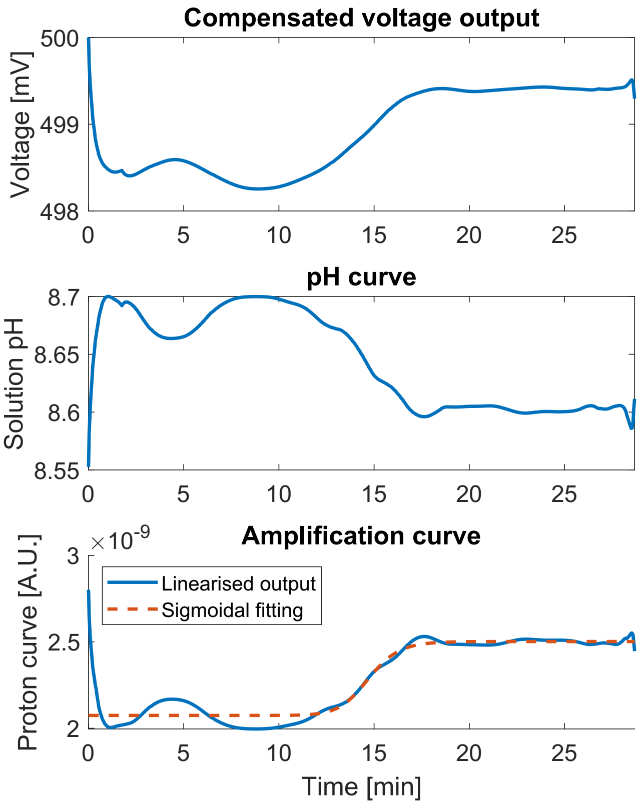

Figure S4. H7.

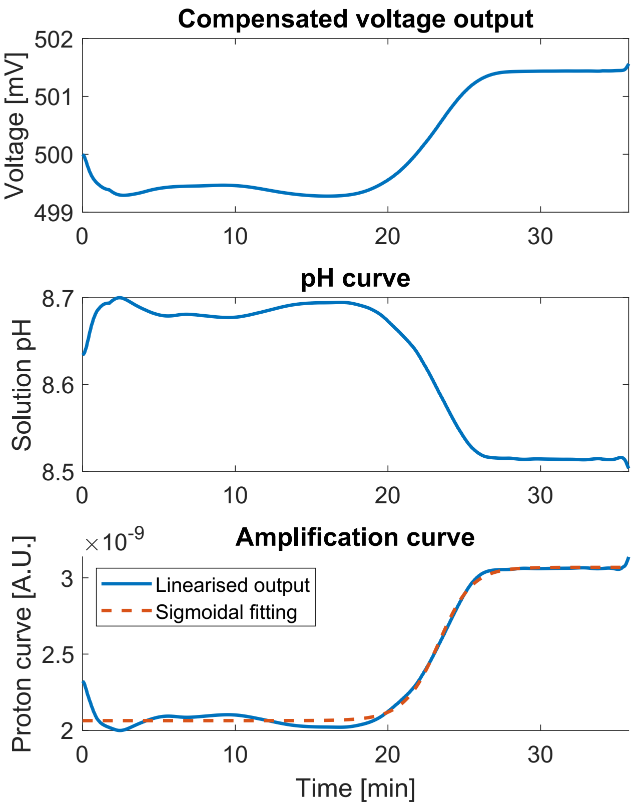

Figure S5. H7 hTERT.

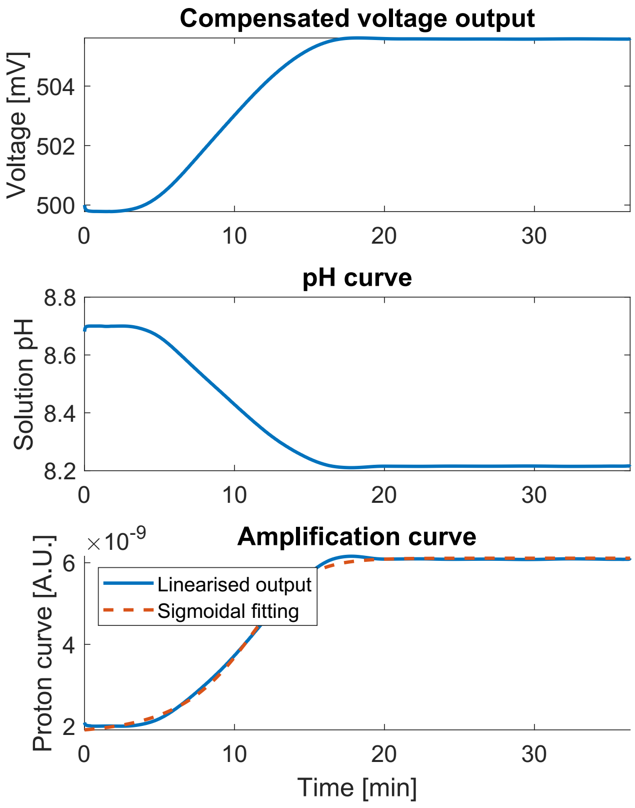

Figure S6. H11.

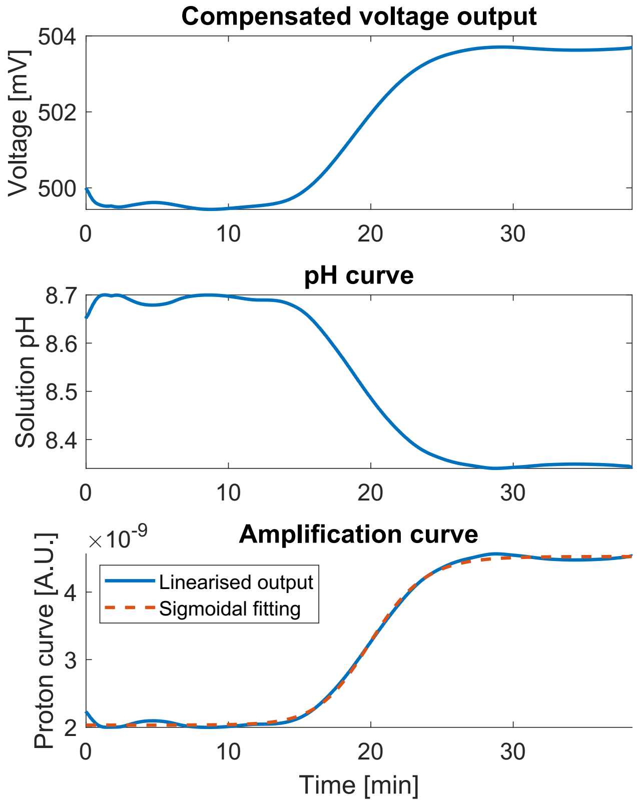

Figure S7. H12.

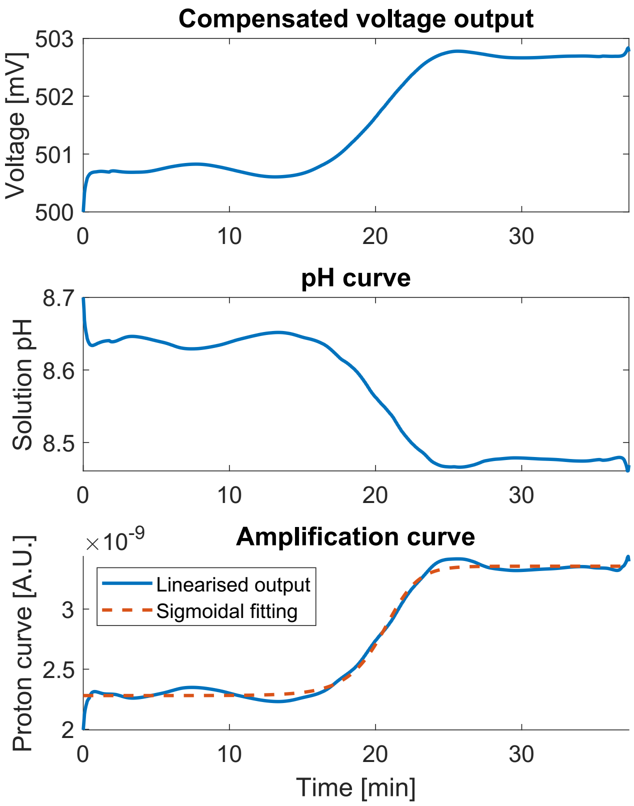

Figure S8. H12 hTERT.

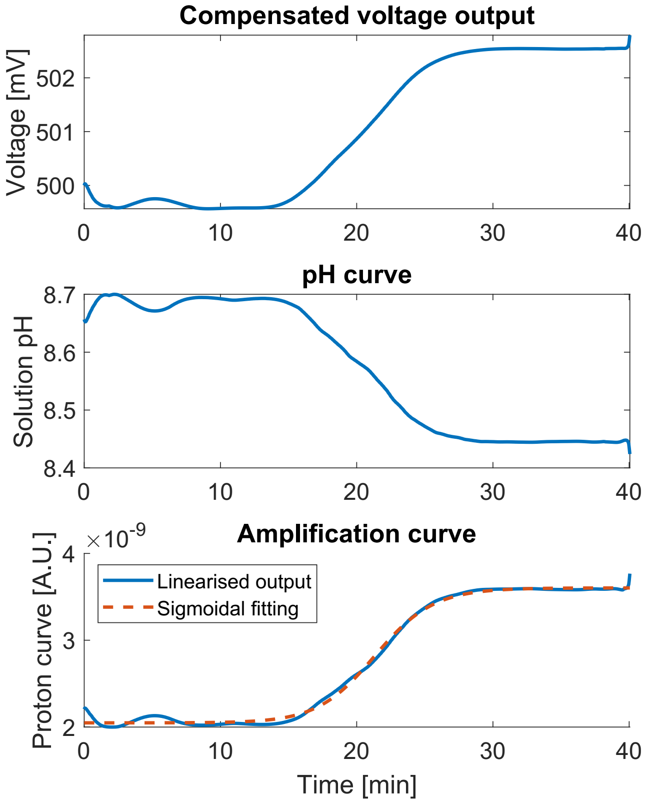

Figure S9. H17.

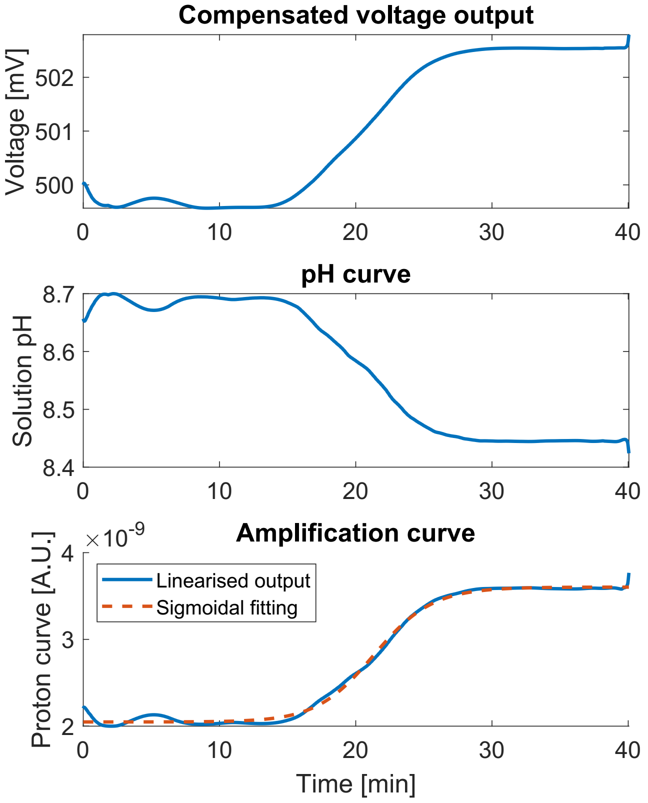

Figure S10. H27.

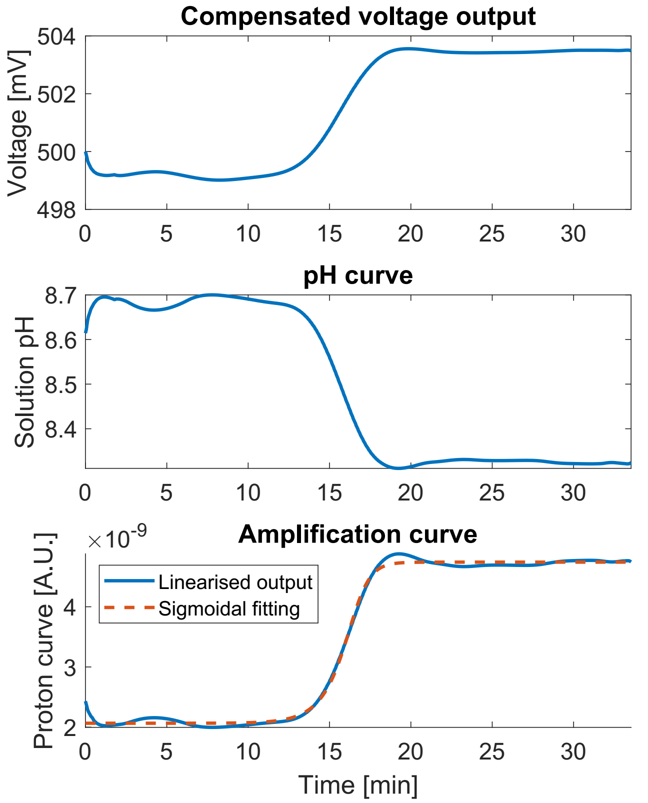

Figure S11. H27 hTERT.

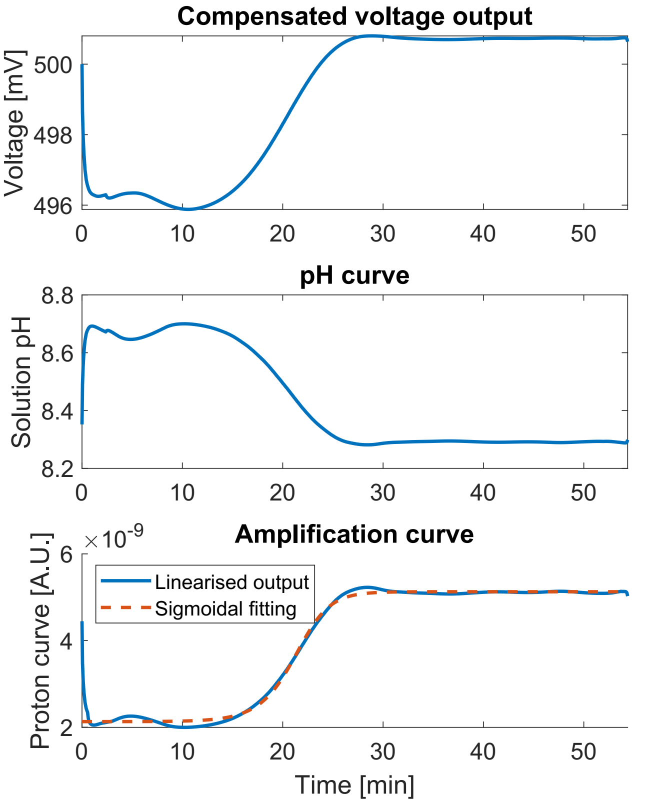

Figure S12. H45.

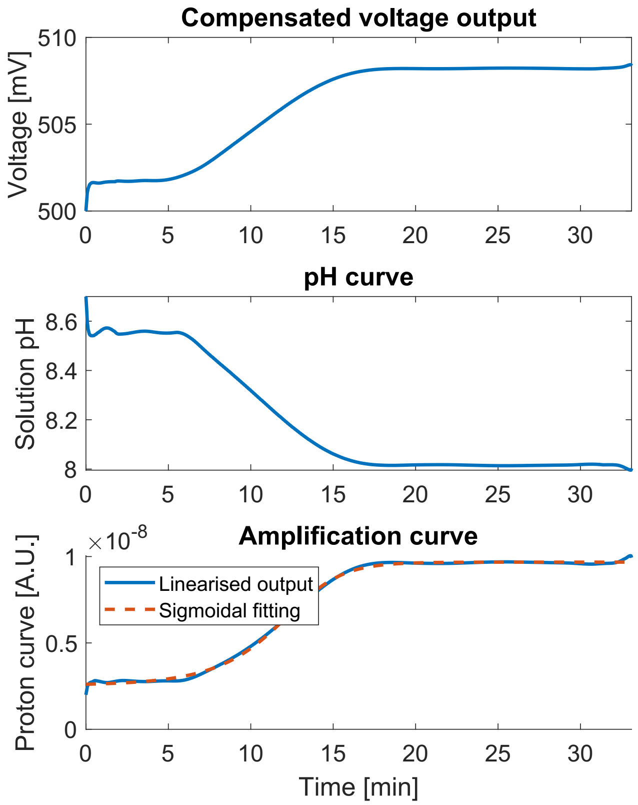

Figure S13. H45 hTERT.
